# Whole-Genome Sequencing of Human Clinical Klebsiella pneumoniae Isolates Reveals Misidentification and Misunderstandings of Klebsiella pneumoniae, Klebsiella variicola, and Klebsiella quasipneumoniae

**DOI:** 10.1128/mSphereDirect.00290-17

**Published:** 2017-08-02

**Authors:** S. Wesley Long, Sarah E. Linson, Matthew Ojeda Saavedra, Concepcion Cantu, James J. Davis, Thomas Brettin, Randall J. Olsen

**Affiliations:** a Center for Molecular and Translational Human Infectious Diseases Research, Department of Pathology and Genomic Medicine, Houston Methodist Research Institute and Houston Methodist Hospital, Houston, Texas, USA; b Department of Pathology and Laboratory Medicine, Weill Cornell Medical College, New York, New York, USA; c Computing, Environment and Life Sciences, Argonne National Laboratory, Argonne, Illinois, USA; d Computation Institute, University of Chicago, Chicago, Illinois, USA; University of Kentucky; Virginia Commonwealth University; Washington University School of Medicine in St. Louis

**Keywords:** clinical microbiology, KPC, Klebsiella pneumoniae, Klebsiella quasipneumoniae, Klebsiella variicola, MALDI, MLST, NDM-1, pathogenesis, whole-genome sequencing, bioinformatics, clinical methods

## Abstract

Klebsiella pneumoniae is a major threat to public health, causing significant morbidity and mortality worldwide. The emergence of highly drug-resistant strains is particularly concerning. There has been a recognition and division of Klebsiella pneumoniae into three distinct phylogenetic groups: Klebsiella pneumoniae, Klebsiella variicola, and Klebsiella quasipneumoniae. *K. variicola* and *K. quasipneumoniae* have often been described as opportunistic pathogens that have less virulence in humans than K. pneumoniae does. We recently sequenced the genomes of 1,777 extended-spectrum-beta-lactamase (ESBL)-producing K. pneumoniae isolates recovered from human infections and discovered that 28 strains were phylogenetically related to *K*. *variicola* and *K. quasipneumoniae*. Whole-genome sequencing of 95 additional non-ESBL-producing K. pneumoniae isolates recovered from patients found 12 *K. quasipneumoniae* strains. Matrix-assisted laser desorption ionization–time of flight mass spectrometry (MALDI-TOF MS) analysis initially identified all patient isolates as K. pneumoniae, suggesting a potential pitfall in conventional clinical microbiology laboratory identification methods. Whole-genome sequence analysis revealed extensive sharing of core gene content and plasmid replicons among the *Klebsiella* species. For the first time, strains of both *K. variicola* and *K. quasipneumoniae* were found to carry the Klebsiella pneumoniae carbapenemase (KPC) gene, while another *K. variicola* strain was found to carry the New Delhi metallo-beta-lactamase 1 (NDM-1) gene. *K. variicola* and *K. quasipneumoniae* infections were not less virulent than K. pneumoniae infections, as assessed by in-hospital mortality and infection type. We also discovered evidence of homologous recombination in one *K. variicola* strain, as well as one strain from a novel *Klebsiella* species, which challenge the current understanding of interrelationships between clades of *Klebsiella*.

**IMPORTANCE** Klebsiella pneumoniae is a serious human pathogen associated with resistance to multiple antibiotics and high mortality. *K. variicola* and *K. quasipneumoniae* are closely related organisms that are generally considered to be less-virulent opportunistic pathogens. We used a large, comprehensive, population-based strain collection and whole-genome sequencing to investigate infections caused by these organisms in our hospital system. We discovered that *K. variicola* and *K. quasipneumoniae* isolates are often misidentified as K. pneumoniae by routine clinical microbiology diagnostics and frequently cause severe life-threatening infections similar to K. pneumoniae. The presence of KPC in *K. variicola* and *K. quasipneumoniae* strains as well as NDM-1 metallo-beta-lactamase in one *K. variicola* strain is particularly concerning because these genes confer resistance to many different beta-lactam antibiotics. The sharing of plasmids, as well as evidence of homologous recombination, between these three species of *Klebsiella* is cause for additional concern.

## Abstract (precis)

Klebsiella pneumoniae is a serious human pathogen associated with resistance to multiple antibiotics and high mortality. *K. variicola* and *K. quasipneumoniae* are closely related organisms that are generally considered to be less-virulent opportunistic pathogens. We used a large, comprehensive, population-based strain collection and whole-genome sequencing to investigate infections caused by these organisms in our hospital system. We discovered that *K. variicola* and *K. quasipneumoniae* isolates are often misidentified as K. pneumoniae by routine clinical microbiology diagnostics and frequently cause severe life-threatening infections similar to K. pneumoniae. The presence of KPC in *K. variicola* and *K. quasipneumoniae* strains as well as NDM-1 metallo-beta-lactamase in one *K. variicola* strain is particularly concerning because these genes confer resistance to many different beta-lactam antibiotics. The sharing of plasmids, as well as evidence of homologous recombination, between these three species of *Klebsiella* is cause for additional concern.

## INTRODUCTION

Edwin Klebs first described Klebsiella pneumoniae organisms in 1875 while examining the airways of patients who died from pneumonia, and Carl Friedlander formally described the species in 1882 (1). Ever since, Klebsiella pneumoniae has been increasingly recognized as a cause of significant human morbidity and mortality (2, 3). Of great concern to public health, many community-acquired and health care-associated outbreaks of invasive K. pneumoniae disease have been reported (4, 5). Neonates, the elderly, and immunocompromised individuals are at greatest risk for poor outcomes (5). The ability of this organism, and other closely related species, to undergo chromosomal recombination and exchange plasmids enables them to readily alter the repertoire of virulence factors and antimicrobial resistance genes (6).

Over the past 2 decades, many related *Klebsiella* species have been identified and classified (7–10). Previously referred to as phylogroups KpII and KpIII, Klebsiella quasipneumoniae (10) and Klebsiella variicola (9) likely diverged from a common ancestor of K. pneumoniae six million years ago (11, 12). Whereas *K. quasipneumoniae* is typically recovered from the gastrointestinal tracts of healthy humans, *K. variicola* organisms are frequently isolated from agricultural sources such as plants, surface water, sewage, soil, and mucosal surfaces of plant-eating livestock (7, 13, 14). Recovery of *K. quasipneumoniae* and *K*. *variicola* strains from patients is generally thought to be representative of colonization (8, 10); however, opportunistic infections have been reported (15–17). The pathogenicity of these organisms in human infection has not been thoroughly studied.

The objective of this study was to investigate the population genetic structure of *K. variicola* and *K. quasipneumoniae* strains recovered from human infections in our health care system (Houston Methodist Hospital System). Whole-genome sequencing demonstrated that K. pneumoniae, *K. quasipneumoniae*, and *K. variicola* strains share chromosomal and mobile genes encoding virulence factors and antimicrobial resistance mechanisms. Importantly, we identified for the first time *K. variicola* strains carrying the Klebsiella pneumoniae carbapenemase (KPC) and New Delhi metallo-beta-lactamase 1 (NDM-1) antimicrobial resistance genes, as well as a *K. quasipneumoniae* strain carrying the KPC antimicrobial resistance gene. Furthermore, we describe the misidentification of *K. variicola* and *K. quasipneumoniae* as K. pneumoniae by matrix-assisted laser desorption ionization—time of flight mass spectrometry (MALDI-TOF MS) and low-resolution sequence typing. We provide detail on the diversity and severity of human infections caused by these pathogens, including one novel strain related to *K. variicola*. These data provide new insight into the pathogenesis of the underrecognized human pathogens *K. quasipneumoniae* and *K. variicola*.

## RESULTS

### Whole-genome sequencing reveals that *K. variicola* and *K. quasipneumoniae* are misidentified by MALDI-TOF MS as K. pneumoniae.

**FIG 1:**
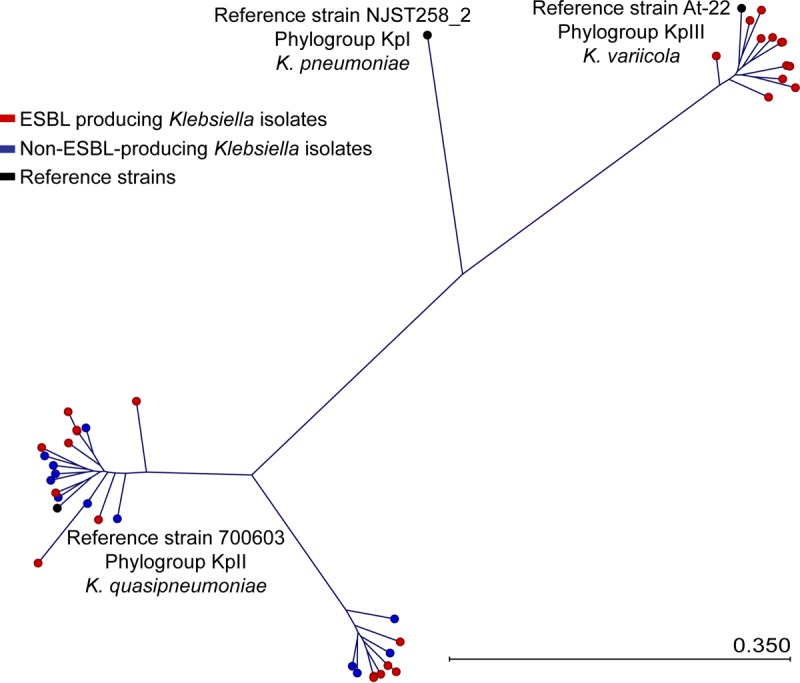
Phylogenetic tree of *K. variicola* and *K. quasipneumoniae* from human infections. Polymorphisms were called against the K. pneumoniae reference genome NJST258_2 (KpI). ESBL-producing *Klebsiella* isolates are represented with red circles, while non-ESBL-producing *Klebsiella* are represented with blue circles. The *K. variicola* clade (KpIII) is at the top right, with the At-22 reference strain indicated by a black circle. The two clades of *K. quasipneumoniae* (KpII) are present at the bottom left, with the reference genome 700603 indicated by the black circle. The outlier *Klebsiella* KPN1705 strain is not shown. The scale bar indicates the mean number of nucleotide substitutions per site.

To study the population genetic structure of Klebsiella pneumoniae isolates recovered from patients in our health care system (Houston Methodist Hospital System) between 2011 and 2015, we recently sequenced the genomes of 1,777 extended-spectrum-beta-lactamase (ESBL)-producing strains (18). All isolates in this comprehensive population-based collection were identified as K. pneumoniae using MALDI-TOF MS in our diagnostic microbiology laboratory (19, 20). We unexpectedly discovered that 28 strains were phylogenetically allied with *K*. *variicola* (13 strains) and *K. quasipneumoniae* (15 strains) (Table 1 and Fig. 1). Thus, in our health care system, ESBL-producing *K. variicola* and *K. quasipneumoniae* cause approximately 2% of human infections attributed to K. pneumoniae. To determine the possible additional presence of *K. variicola* and *K. quasipneumoniae* among non-ESBL-producing K. pneumoniae, we sequenced the genomes of 95 strains recovered in 2017. We discovered that 12 non-ESBL-producing strains were also phylogenetically allied with *K*. *quasipneumoniae* (12.6% of non-ESBL-producing isolates) (Table 1 and Fig. 1). The *K. variicola* and *K. quasipneumoniae* strains were recovered from multiple anatomic sites, including blood, drains, respiratory specimens, tissue, and urine (Table 1).

**TABLE 1:** Summary of the clinical characteristics of the *K. variicola* and *K. quasipneumoniae* strains

Strain	MALDI-TOF MS identification	Genomic identification	ESBL producing	Collection date (mo/yr)	Sample source	Associated patient mortality
KPN325	K. pneumoniae	*K. variicola*	Yes	6/2012	Urine	No
KPN349	K. pneumoniae	*K. variicola*	Yes	7/2012	Wound	No
KPN458	K. pneumoniae	*K. variicola*	Yes	10/2012	Blood	No
KPN700	K. pneumoniae	*K. variicola*	Yes	3/2013	Blood	No
KPN771	K. pneumoniae	*K. variicola*	Yes	5/2013	Drain	No
KPN807	K. pneumoniae	*K. variicola*	Yes	6/2013	Urine	No
KPN1264	K. pneumoniae	*K. variicola*	Yes	2/2014	Respiratory	No
KPN1401	K. pneumoniae	*K. variicola*	Yes	4/2014	Respiratory	Yes
KPN1415	K. pneumoniae	*K. variicola*	Yes	4/2014	Respiratory	Yes
KPN1481	K. pneumoniae	*K. variicola*	Yes	6/2014	Urine	No
KPN1556	K. pneumoniae	*K. variicola*	Yes	7/2014	Wound	No
KPN1705	K. pneumoniae	*K. variicola*	Yes	8/2014	Wound	No
KPN1751	K. pneumoniae	*K. variicola*	Yes	10/2014	Urine	Yes
KPN560	K. pneumoniae	*K. quasipneumoniae*	Yes	12/2012	Urine	No
KPN712	K. pneumoniae	*K. quasipneumoniae*	Yes	4/2013	Respiratory	No
KPN1132	K. pneumoniae	*K. quasipneumoniae*	Yes	12/2013	Urine	No
KPN1398	K. pneumoniae	*K. quasipneumoniae*	Yes	4/2014	Respiratory	No
KPN1470	K. pneumoniae	*K. quasipneumoniae*	Yes	5/2014	Drain	No
KPN1533	K. pneumoniae	*K. quasipneumoniae*	Yes	8/2014	Urine	No
KPN1648	K. pneumoniae	*K. quasipneumoniae*	Yes	9/2014	Wound	No
KPN1673	K. pneumoniae	*K. quasipneumoniae*	Yes	9/2014	Wound	No
KPN1688	K. pneumoniae	*K. quasipneumoniae*	Yes	8/2014	Blood	Yes
KPN1711	K. pneumoniae	*K. quasipneumoniae*	Yes	9/2014	Wound	No
KPN1715	K. pneumoniae	*K. quasipneumoniae*	Yes	10/2014	Respiratory	No
KPN1962	K. pneumoniae	*K. quasipneumoniae*	Yes	2/2015	Urine	No
KPN2096	K. pneumoniae	*K. quasipneumoniae*	Yes	3/2015	Urine	No
KPN2105	K. pneumoniae	*K. quasipneumoniae*	Yes	4/2015	Drain	No
KPN2119	K. pneumoniae	*K. quasipneumoniae*	Yes	4/2015	Urine	Yes
NEK11	K. pneumoniae	*K. quasipneumoniae*	No	2/2017	Urine	No
NEK12	K. pneumoniae	*K. quasipneumoniae*	No	2/2017	Urine	No
NEK19	K. pneumoniae	*K. quasipneumoniae*	No	2/2017	Urine	No
NEK36	K. pneumoniae	*K. quasipneumoniae*	No	2/2017	Urine	No
NEK42	K. pneumoniae	*K. quasipneumoniae*	No	2/2017	Urine	No
NEK47	K. pneumoniae	*K. quasipneumoniae*	No	2/2017	Urine	No
NEK56	K. pneumoniae	*K. quasipneumoniae*	No	2/2017	Blood	No
NEK59	K. pneumoniae	*K. quasipneumoniae*	No	2/2017	Respiratory	No
NEK66	K. pneumoniae	*K. quasipneumoniae*	No	2/2017	Urine	No
NEK67	K. pneumoniae	*K. quasipneumoniae*	No	2/2017	Urine	No
NEK118	K. pneumoniae	*K. quasipneumoniae*	No	2/2017	Blood	No
NEK122	K. pneumoniae	*K. quasipneumoniae*	No	3/2017	Urine	No

### Multilocus sequence typing and capsule genotyping misclassify *K. variicola* and *K. quasipneumoniae* isolates as K. pneumoniae.

**FIG 2:**
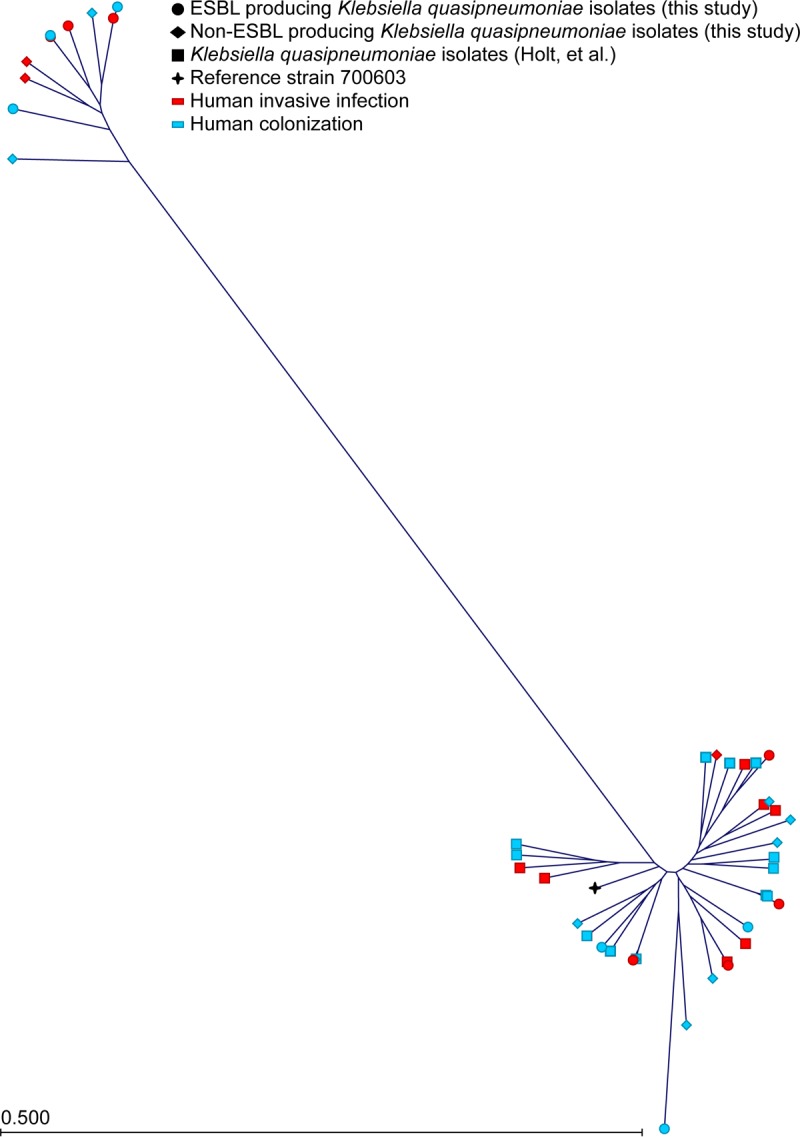
Phylogenetic tree of *K. quasipneumoniae* from humans. Polymorphisms in the *K. quasipneumoniae* strains were called against the 700603 reference genome (black cross). The clade KpIIA is at the top left, while the KpIIB clade is at the bottom right. ESBL-producing strains sequenced for this study are represented by circles. Non-ESBL-producing strains of both clades identified in our collection are represented by diamonds. Strains previously sequenced by Holt et al. (8) are represented by squares. Strains associated with human invasive infection sites are indicated in red, and human colonization is indicated in blue.

**FIG 3:**
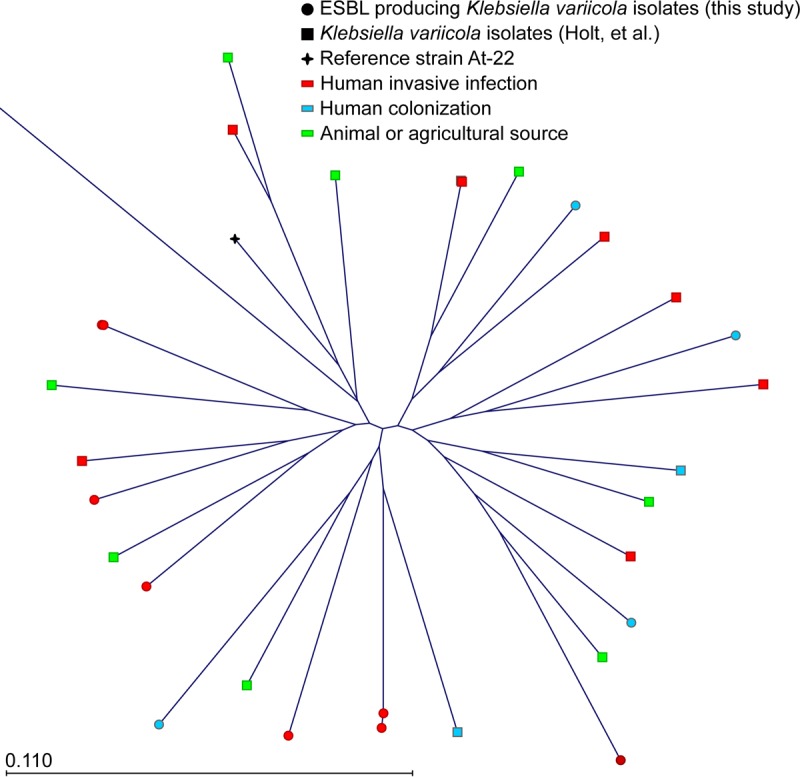
Phylogenetic tree of *K. variicola* from humans and animals. Polymorphisms in the *K. variicola* strains were called against the At-22 reference genome (black cross). All *K. variicola* isolates identified were ESBL producers. There is one outlier *Klebsiella*, KPN1705, which projects beyond the border of the figure to the top left. Strains sequenced for this study are represented by circles, while those previously sequenced by Holt et al. (8) are represented by squares. Strains associated with human invasive infection sites are indicated in red, human colonization is indicated in blue, and animal-associated isolates are indicated in green.

Over the years, *K. variicola* and *K. quasipneumoniae* strains have been misclassified as K. pneumoniae, resulting in the deposition of misidentified reference strains in public repositories, including the public K. pneumoniae multilocus sequence typing (MLST) schema (21–24). We used the whole-genome sequence data to generate a K. pneumoniae MLST assignment for each strain. In our collection of 13 ESBL-producing *K. variicola* isolates, 6 (46%) had a defined K. pneumoniae MLST type and 3 (23%) had a novel combination of known K. pneumoniae MLST alleles (Table 2). Among the 15 ESBL-producing *K. quasipneumoniae* isolates, 12 (80%) had a defined K. pneumoniae MLST type, and 1 (7%) had a novel combination of known alleles (Table 2). Similarly, among the 12 non-ESBL-producing *K*. *quasipneumoniae* strains, 3 (25%) had a defined K. pneumoniae MLST type, and 6 (50%) had a novel combination of known alleles (Table 2). Consistent with the extensive genetic diversity observed within each *Klebsiella* clade (Fig. 2 and 3), multiple different MLST designations were found for *K. variicola* and *K. quasipneumoniae* strains in our collection (Table 2).

**TABLE 2:** Genomic characteristics of the *K. variicola* and *K. quasipneumoniae* strains^a^

Strain	Species	MLST	Capsule	*nif*	KPC	CTX-M	NDM-1	SHV-OKP- LEN core	SHV-OKP- LEN plasmid	TEM
KPN325	*K. variicola*	1174	KL33*	+	−	−	−	LEN-24	SHV-12	−
KPN349	*K. variicola*	468	KL60	+	−	−	−	LEN-24	SHV-5	−
KPN458	*K. variicola*	NF	KL19	+	−	−	−	LEN-24	SHV-12	−
KPN700	*K. variicola*	681	KL143*	+	−	−	−	LEN-2	−	−
KPN771	*K. variicola*	681	KL143*	+	−	−	−	LEN-2	−	−
KPN807	*K. variicola*	NF	KL105	+	−	CTX-M-15	−	OKP-B-16	−	TEM-166
KPN1264	*K. variicola*	454	KL109*	−	−	−	−	LEN-24	SHV-30	−
KPN1401	*K. variicola*	NF	KL19	+	KPC-2	−	−	LEN-20	−	−
KPN1415	*K. variicola*	NF	KL19	+	KPC-2	−	−	LEN-4	−	TEM-79
KPN1481	*K. variicola*	906	KL10	+	−	CTX-M-15	NDM-1	LEN-16	−	−
KPN1556	*K. variicola*	NF	KL53*	+	−	−	−	LEN-24	SHV-12	−
KPN1705	*K. variicola*	NF	KL153	+	−	−	−	LEN-24	SHV-30	−
KPN1751	*K. variicola*	1456	KL60	+	−	−	−	LEN-24	SHV-12, SHV-66	−
KPN560	*K. quasipneumoniae*	1602	KL53	−	−	CTX-M-15	−	OKP-B-19		TEM-198
KPN712	*K. quasipneumoniae*	NF	KL101	−	−	−	−	OKP-B-3	SHV-12	−
KPN1132	*K. quasipneumoniae*	476	KL57	−	−	−	−	OKP-B-15	SHV-12	−
KPN1398	*K. quasipneumoniae*	2133	KL106*	+	−	CTX-M-15	−	OKP-B-15	−	TEM-166
KPN1470	*K. quasipneumoniae*	138	KL1	+	−	CTX-M-15	−	OKP-B-2	−	TEM-33
KPN1533	*K. quasipneumoniae*	2351	KL114	+	−	−	−	OKP-A-5	SHV-12	−
KPN1648	*K. quasipneumoniae*	2351	KL114	+	−	CTX-M-3	−	OKP-A-5	−	TEM-30
KPN1673	*K. quasipneumoniae*	1887	KL121	+	−	−	−	OKP-B-8	SHV-12	−
KPN1688	*K. quasipneumoniae*	1887	KL121	+	−	−	−	OKP-B-8	SHV-12	−
KPN1711	*K. quasipneumoniae*	NF	KL1	+	KPC-2	−	−	OKP-A-7	−	−
KPN1715	*K. quasipneumoniae*	1887	KL121*	+	−	−	−	OKP-B-8	SHV-12	−
KPN1962	*K. quasipneumoniae*	196	KL46	−	−	−	−	OKP-A-5	−	TEM-198
KPN2096	*K. quasipneumoniae*	414	KL123	−	−	−	−	OKP-B-8	−	−
KPN2105	*K. quasipneumoniae*	978	KL125	+	−	CTX-M-3	−	OKP-A-3	−	−
KPN2119	*K. quasipneumoniae*	978	KL125	+	−	CTX-M-3	−	OKP-A-3	−	−
NEK11	*K. quasipneumoniae*	138	KL1	+	−	−	−	OKP-B-2	−	−
NEK12	*K. quasipneumoniae*	NF	KL16	+	−	−	−	OKP-B-5	−	−
NEK19	*K. quasipneumoniae*	NF	KL16	+	−	−	−	OKP-B-3	−	TEM-198
NEK36	*K. quasipneumoniae*	283	KL10	+	−	−	−	OKP-B-15	−	−
NEK42	*K. quasipneumoniae*	NF	KL33	+	−	−	−	OKP-A-11	−	−
NEK47	*K. quasipneumoniae*	NF	KL158	+	−	−	−	OKP-A-7	−	−
NEK56	*K. quasipneumoniae*	894	KL58	−	−	−	−	OKP-B-15	−	−
NEK59	*K. quasipneumoniae*	NF	KL128	+	−	−	−	OKP-A-10	−	−
NEK66	*K. quasipneumoniae*	NF	KL139	−	−	−	−	OKP-B-1	−	−
NEK67	*K. quasipneumoniae*	477	KL15	+	−	−	−	OKP-B-3	−	−
NEK118	*K. quasipneumoniae*	NF	KL13	+	−	−	−	OKP-A-11	−	−
NEK122	*K. quasipneumoniae*	NF	KL56	+	−	−	−	OKP-B-5	−	−

a The presence or absence of select antimicrobial resistance genes, MLST, capsule genotype, presence or absence of *nif* genes, and presence of SHV-LEN-OKP beta-lactamase in core genome and plasmids. Capsule loci that appear to be variants are indicated with an asterisk.

Capsule serotype has been used for close to a century to classify *Klebsiella* species in general (25). Capsule genotype, as assessed by sequencing the genes in the capsule locus, has been used more recently as a molecular tool to subclassify K. pneumoniae strains (26–28). Among our collection of *K. variicola* and *K. quasipneumoniae* isolates, every strain had a K. pneumoniae capsule locus detected (Table 2). Again, consistent with the extensive genetic diversity observed within each clade, multiple different capsule genotypes were found, including some strains with novel combinations of capsule genes (Table 2). Taken together, these data demonstrate that the majority of *K. variicola* and *K. quasipneumoniae* strains recovered from human patients would be misidentified by typing methods such as K. pneumoniae MLST and capsule genotyping.

### Whole-genome sequencing reveals extensive genetic diversity within and between *Klebsiella* clades.

To begin assessing phylogenetic relationships between K. pneumoniae, *K. variicola*, and *K. quasipneumoniae* strains, single nucleotide polymorphisms (SNPs) were determined relative to the ST258 K. pneumoniae reference genome NJST258_2 (GenBank accession number CP006918.1) (6). Consistent with a previous report using strains collected from a variety of human, animal, and environmental sources (8), each *Klebsiella* species formed a distinct clade, with *K. quasipneumoniae* divided into two subclades (Fig. 1). Phylogenetic analysis demonstrated that the ESBL-producing and non-ESBL-producing *K. quasipneumoniae* strains are intermingled on the cladogram, suggesting that they are derived from a common genetic pool (Fig. 2). However, this does not imply that our ESBL-producing and non-ESBL-producing *K. quasipneumoniae* strains are identical, because both groups include strains with a diverse array of different MLST, capsule, and plasmid genotypes (Tables 2 and 3).

Phylogenetic comparison of our *K*. *quasipneumoniae* isolates recovered from infected patients with publicly available sequence data from strains collected from humans (8) showed much genetic intermixing (Fig. 2). Similarly, analysis of *K. variicola* strains recovered from our patients with invasive infection (Table 1) or colonized humans and animals (8) also revealed no clear phylogenetic segregation (Fig. 3). Taken together, these data suggest that *K. quasipneumoniae* and *K. variicola* strains recovered from infected patients and colonized humans or animals are derived from a common genetic pool.

One outlier strain, KPN1705, was present in the *K. variicola* population (Fig. 3). The KPN1705 strain was allied phylogenetically with *K. variicola*, yet it was 251,390 SNPs distant from the *K. variicola* At-22 reference genome. This was striking compared to the average distance to the At-22 reference of 38,056 SNPs (range, 31,777 to 45,299) in our other *K. variicola* strains. Since hybrid strains of *Klebsiella* with large chromosomal recombination events have been described previously (8), we compared the reads from strain KPN1705 against At-22 (*K. variicola*), NJST258_2 (K. pneumoniae), and 700603 (*K. quasipneumoniae*) reference genomes. The SNP distribution was uniform across the three reference genomes, which made the possibility of KPN1705 being the result of a single large recombination event less likely (data not shown). To further evaluate its phylogeny, we determined that KPN1705 was a single locus variant of the K. pneumoniae ST1155. The only other ST1155 strain reported in the literature is designated 10982 and is proposed to represent a novel species (22). Strain 10982 was isolated from a perianal swab from an intensive care unit (ICU) patient in Maryland in 2005. Thus, our strain KPN1705 appears most closely related to a novel *Klebsiella* species that colonizes the gastrointestinal (GI) tract of humans and may be capable of causing human disease.

### SHV-LEN-OKP core chromosomal beta-lactamases are not restricted by *Klebsiella* species.

The SHV-LEN-OKP beta-lactamases are core chromosomal genes of *Klebsiella* that have been suggested to be differentiators of *Klebsiella* species: K. pneumoniae (SHV restricted), *K. quasipneumoniae* (OKP restricted), and *K. variicola* (LEN restricted) (10, 29, 30). However, one potential confounder is that the SHV beta-lactamase genes can also be carried on plasmids (31). We next used the whole-genome sequence data to assess SHV-LEN-OKP gene content in our 40 *K. variicola* and *K. quasipneumoniae* strains. Among our collection, 7/13 (54%) *K. variicola* strains carried SHV on plasmids in addition to the chromosomal LEN gene (Table 2). Similarly, 7/15 (47%) *K. quasipneumoniae* strains carried SHV on a plasmid in addition to the chromosomal OKP gene. Only 1/12 (8%) non-ESBL-producing *K*. *quasipneumoniae* isolate carried SHV on a plasmid in addition to the chromosomal OKP genes. The presence of SHV on plasmids, sometimes in multiple copies, may complicate the identification and analysis of *Klebsiella* strains.

**FIG 4:**
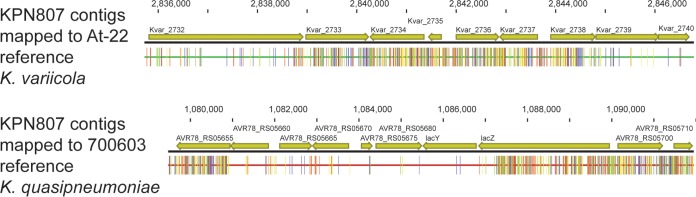
Comparison of the 7-kb region of recombination in strain KPN807. KPN807 assembled contigs are compared to the At-22 K. *variicola* reference genome (top) and the 700603 *K. quasipneumoniae* reference genome (bottom). The consensus is indicated by a red bar, with SNPs marked as colored vertical lines. KPN807 has a marked increase in SNP density in the center of the region relative to At-22 and the flanking regions relative to 700603, suggesting that this 7-kb region of the chromosome has recombined into the *K. variicola* KPN807 from a *K. quasipneumoniae* isolate.

Unexpectedly, we discovered one *K. variicola* strain (designated KPN807; Table 2) carrying a chromosomal copy of OKP rather than the expected LEN gene that is typical of *K. variicola* strains. This OKP gene was carried within a 7-kb segment of chromosomal DNA that had a higher sequence identity to *K. quasipneumoniae* than *K. variicola*, suggesting recombination of this region (Fig. 4).

### *K. variicola* and *K. quasipneumoniae* have similar antimicrobial resistance and plasmid replicon content to K. pneumoniae, including KPC and NDM-1.

**FIG 5:**
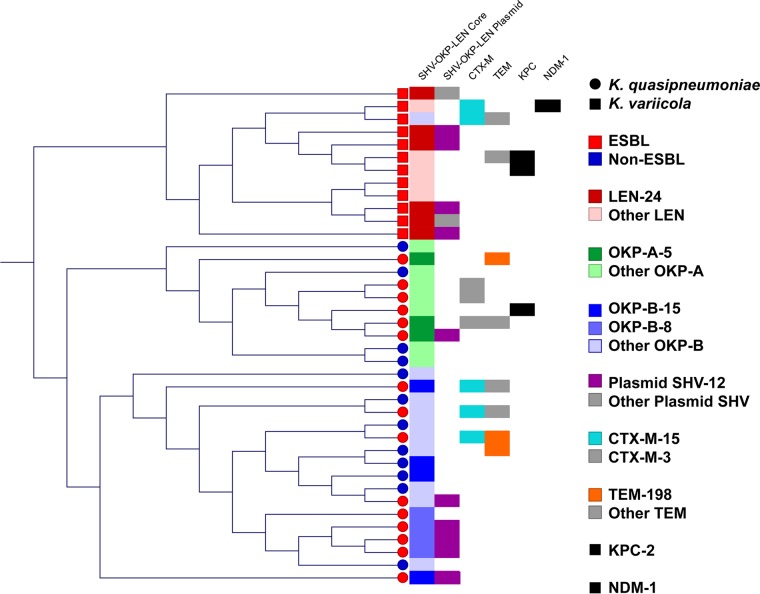
Beta-lactamase gene content of K. pneumoniae, *K. variicola*, and *K. quasipneumoniae*. The phylogenetic relationship between strains based upon the presence of beta-lactamase genes is shown on a rectangular cladogram. Polymorphisms were called against the K. pneumoniae reference genome NJST258_2. *K. quasipneumoniae* are represented by circles, while *K. variicola* are represented by squares. ESBL-producing *Klebsiella* are shown in red, while non-ESBL producing *Klebsiella* are shown in blue. To the right of the cladogram, the common beta-lactamase genes are shown. The first column lists the core genome SHV-OKP-LEN beta-lactamase: LEN in red, OKP-A in green, and OKP-B in blue. No SHV was found in the core chromosome. The most common allele(s) for OKP and LEN is represented with a darker shade of the primary color. The second column represents the plasmid SHV-OKP-LEN beta-lactamase content, with the most common allele SHV-12 in purple and all others in gray. The third column indicates the CTX-M-15 alleles in teal, with CTX-M-3 in gray. The fourth column shows the TEM alleles, with the most common allele TEM-198 in orange and all others in gray. The fifth and sixth columns represent the presence of KPC-2 and NDM-1 alleles in black, respectively.

The emergence of multidrug resistance among K. pneumoniae strains is a cause of public concern. A table of the MIC values for the 40 *K. variicola* and *K. quasipneumoniae* strains is provided in Table S1 in the supplemental material. Next, we used the whole-genome sequence data to compare plasmid replicons carried by the different *Klebsiella* species. There were many plasmid replicons and antimicrobial resistance genes among the ESBL-producing *K. variicola* and *K. quasipneumoniae* isolates that were also identified in K. pneumoniae (Table 3) (18). In general, plasmid replicons tended to be associated with genomically allied strains (Fig. 5). In particular, the FIBk, FIIk, and FII replicons were detected in the majority of our *K*. *variicola* and *K. quasipneumoniae* isolates. Several strains also possessed the KPC-2 carbapenemase and CTX-M beta-lactamase genes associated with multiple drug resistance. Of note, KPC-positive *K. variicola* and *K. quasipneumoniae* strains have not been previously reported in the United States.

10.1128/mSphereDirect.00290-17.1 TABLE S1 MIC testing for the *Klebsiella* strains in this study. Download TABLE S1, XLSX file, 0.1 MB. Copyright © 2017 Long et al. 2017 Long et al. This content is distributed under the terms of the Creative Commons Attribution 4.0 International license.

**TABLE 3:** Plasmid replicons carried by the *K. variicola* and *K. quasipneumoniae* strains^a^

Strain	FIBK_Kpn3	FIIK	FII_1
KPN325	FIBK_1_Kpn3_JN233704_92	_FIIK_1_CP000648	FII_1_pKP91_CP000966_17
KPN349	FIBK_1_Kpn3_JN233704_92	_FIIK_1_CP000648	
KPN458			
KPN700	FIBK_1_Kpn3_JN233704_92		
KPN771			
KPN807	FIBK_1_Kpn3_JN233704_92	_FIIK_1_CP000648	
KPN1264	FIBK_1_Kpn3_JN233704_92	FIIK_2_CP000966_pKp91_67	FII_1_pKP91_CP000966_17
KPN1401		_FIIK_1_CP000648	FII_1_pSFO_AF401292_16
KPN1415		_FIIK_1_CP000648	FII_3_AF401292_pSFo157_18
KPN1481		FIIK_2_CP000966_pKp91_67	FII_1_pKP91_CP000966_17
KPN1556	FIBK_1_Kpn3_JN233704_92	_FIIK_1_CP000648	FII_1_pKP91_CP000966_17
KPN1705	FIBK_1_Kpn3_JN233704_92	_FIIK_1_CP000648	
KPN1751	FIBK_1_Kpn3_JN233704_92	_FIIK_1_CP000648	FII_1_pKP91_CP000966_17
KPN560			
KPN712			
KPN1132	FIBK_1_Kpn3_JN233704_92		
KPN1398	FIBK_1_Kpn3_JN233704_92	_FIIK_1_CP000648	FII_1_pKP91_CP000966_17
KPN1470	FIBK_1_Kpn3_JN233704_92	_FIIK_1_CP000648	
KPN1533	FIBK_1_Kpn3_JN233704_92	_FIIK_1_CP000648	FII_1_pKP91_CP000966_17
KPN1648	FIBK_1_Kpn3_JN233704_92		
KPN1673	FIBK_1_Kpn3_JN233704_92		
KPN1688	FIBK_1_Kpn3_JN233704_92		
KPN1711	FIBK_1_Kpn3_JN233704_92	FIIK_2_CP000966_pKp91_67*	FII_1_pKP91_CP000966_17
KPN1715	FIBK_1_Kpn3_JN233704_92		
KPN1962	FIBK_1_Kpn3_JN233704_92		FII29_1_pUTI89_CP003035_15
KPN2096	FIBK_1_Kpn3_JN233704_92	_FIIK_1_CP000648	
KPN2105		_FIIK_1_CP000648	
KPN2119	FIBK_1_Kpn3_JN233704_92	_FIIK_1_CP000648	FII_1_pKP91_CP000966_17
NEK11	FIBK_1_Kpn3_JN233704_92		
NEK12	FIBK_1_Kpn3_JN233704_92		
NEK19	FIBK_1_Kpn3_JN233704_92	_FIIK_1_CP000648	
NEK36	FIBK_1_Kpn3_JN233704_92	_FIIK_1_CP000648	FII_1_pKP91_CP000966_17
NEK42		_FIIK_1_CP000648	
NEK47	FIBK_1_Kpn3_JN233704_92		
NEK56	FIBK_1_Kpn3_JN233704_92	_FIIK_1_CP000648	
NEK59	FIBK_1_Kpn3_JN233704_92		
NEK66	FIBK_1_Kpn3_JN233704_92		
NEK67	FIBK_1_Kpn3_JN233704_92	_FIIK_1_CP000648	FII_1_pKP91_CP000966_17
NEK118	FIBK_1_Kpn3_JN233704_92		
NEK122			

a Presence or absence of the three most common plasmid replicons found in the *Klebsiella* isolates in this study.

Unexpectedly, one *K. variicola* strain (designated KPN1481; Table 3) carried a plasmid, pKPN1481-1, with the New Delhi metallo-beta-lactamase 1 (NDM-1) gene (GenBank accession numbers CP020847 to CP020852). This represents the first NDM-1-producing *K. variicola* strain to be reported. To better characterize the plasmid and strain containing the NDM-1 gene, it was sequenced to closure using single molecule real-time (SMRT) sequencing. Results confirmed the presence of the NDM-1 gene on pKPN1481-1, a 342-kb plasmid (18). Alignment of pKPN1481-1 to NDM-1 gene-containing plasmids from K. pneumoniae strains recovered from our health care system and elsewhere revealed extensive similarity (18, 32, 33). Importantly, the 10,273-bp region containing the NDM-1 gene was identical in sequence to four NDM-1 plasmids carried by K. pneumoniae isolates in our collection.

### Gene content comparison between K. pneumoniae, *K. variicola*, and *K. quasipneumoniae.*

**FIG 6:**
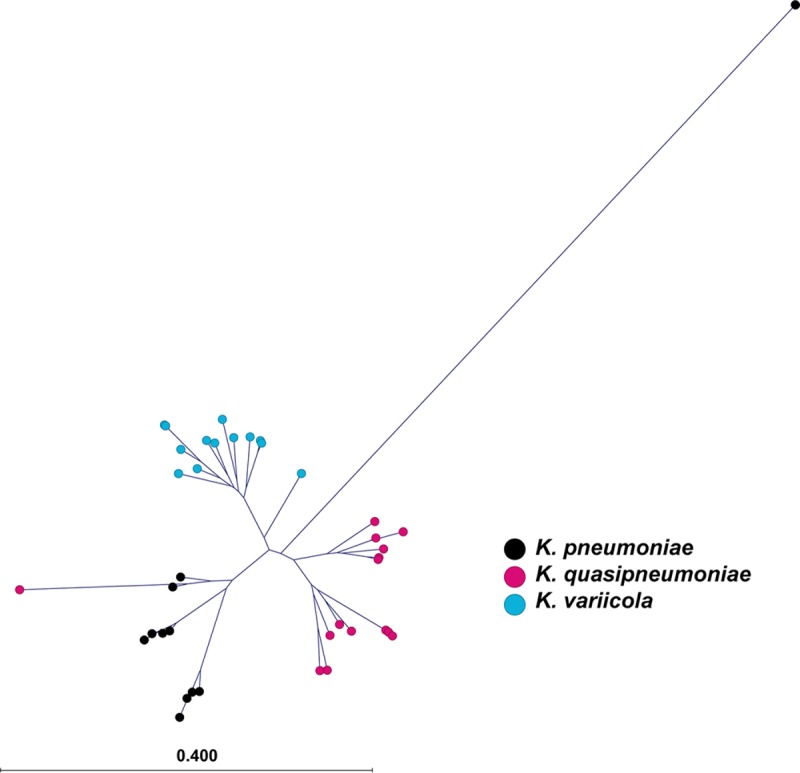
Binary accessory genome tree of K. pneumoniae, *K. variicola*, and *K. quasipneumoniae*. The phylogenetic relationship between strains based upon the presence or absence of genes in the pangenome is shown. The three clades are visible, with the K. pneumoniae strains (black circles), *K. variicola* strains (blue circles), and *K. quasipneumoniae* strains (magenta circles) indicated. The K. pneumoniae strains separate into two clades based upon their MLST type, while the *K. quasipneumoniae* strains separate into the two distinct clades representing *K*. *quasipneumoniae* subsp. *quasipneumoniae* and *K. quasipneumoniae* subsp. *similipneumoniae*. One strain of *K. quasipneumoniae* (KPN1470) is allied with K. pneumoniae, due to similarities in accessory genome content.

We compared the gene content between our ESBL-producing *K. variicola* strains, *K. quasipneumoniae* strains, and 12 representative K. pneumoniae strains from our previously published collection (18). We identified a total of 30,075 unique genes present in the pangenome. A *Klebsiella* core genome was identified consisting of 2,800 unique genes that were present in >95% of the isolates. Most of the accessory genes were present in less than 15% of the isolates (23,334 genes; 77.5% of the total). A binary tree of accessory gene content demonstrates that the accessory gene content is largely differentiated by the three major clades, suggesting a clade-specific core genome for each ESBL *Klebsiella* species as well as the presence of unique mobile genetic content (Fig. 6). This finding is further reinforced when considering the core genome of the *K. variicola* and ESBL-producing *K. quasipneumoniae* in our study. The ESBL-producing *K. quasipneumoniae* strains have a core genome of 3,338 unique genes, while the *K. variicola* strains have a core genome of 3,960 genes. This difference may be in part a reflection of the division of *K. quasipneumoniae* into two distinct clades. The presence of *nif* operon genes, which facilitate nitrogen fixation, has been associated with agricultural isolates of *Klebsiella*. We found *nif* genes present in 12/13 (92.3%) ESBL-producing *K. variicola* human isolates, 11/15 (73.3%) ESBL-producing *K. quasipneumoniae* isolates, and 10/12 (83.3%) non-ESBL-producing *K. quasipneumoniae* isolates, suggesting that *nif* genes may persist in isolates found in human infections*.* The presence or absence of select genes is included in Tables S2 and S3.

10.1128/mSphereDirect.00290-17.2 TABLE S2 Presence or absence of select genes in ESBL-producing *Klebsiella* strains in this study. Download TABLE S2, XLSX file, 4.3 MB. Copyright © 2017 Long et al. 2017 Long et al. This content is distributed under the terms of the Creative Commons Attribution 4.0 International license.

10.1128/mSphereDirect.00290-17.3 TABLE S3 Presence or absence of select genes in non-ESBL-producing *K. quasipneumoniae* strains. Download TABLE S3, XLSX file, 7 MB. Copyright © 2017 Long et al. 2017 Long et al. This content is distributed under the terms of the Creative Commons Attribution 4.0 International license.

### Human infections caused by *K. variicola* and *K. quasipneumoniae* are similar in site and mortality to those caused by K. pneumoniae.

Although a few cases of opportunistic infection have been published, most reports suggest that *K. variicola* and *K. quasipneumoniae* most commonly occupy agricultural niches or benignly colonize the gastrointestinal tract (8, 10, 34). However, among patients in Houston, TX, with infections caused by ESBL-producing organisms, K. pneumoniae, *K. variicola*, and *K. quasipneumoniae* were associated with similar infection types and outcomes (Table 1). That is, ESBL-producing K. pneumoniae, *K. variicola*, and *K. quasipneumoniae* strains caused a similar ratio of invasive infections relative to recovery from the urinary tract (42.5%, 60.0%, and 54.5%, respectively; not significantly different by chi-square test). Also, no significant difference in mortality was observed between the three clades (16.0%, 13.3%, and 18.0%, respectively; not significantly different by the chi-square test).

## DISCUSSION

K. pneumoniae is a well-known cause of life-threatening infections. However, there is conflicting evidence of the pathogenicity of the closely related organisms *K. variicola* and *K. quasipneumoniae*. They are most often associated with agricultural niches (7, 9) or thought to represent colonization rather than infection (8). At present, *K. variicola* and *K. quasipneumoniae* are viewed most commonly as benign endosymbionts of plants, colonizers of the gastrointestinal tracts of animals and humans that consume these foods, or occasional opportunistic pathogens. However, a recent report from Stockholm, Sweden, suggests that these organisms may be an underrecognized cause of bacteremia, including fatal infections (23). Among patients at our health care system, *K. variicola* and *K. quasipneumoniae* strains caused a minority of *Klebsiella* infections. However, when ESBL-producing *K. variicola* and *K. quasipneumoniae* infections did occur, they were as virulent as ESBL-producing K. pneumoniae strains, causing invasive infections and mortality at rates statistically similar to those of K. pneumoniae strains.

Our whole-genome sequence data provide clues to the similar virulence of *K. variicola* and *K. quasipneumoniae* strains relative to K. pneumoniae strains. The core genome contents of strains in the three clades are similar, despite the extent of single nucleotide polymorphism and divergence between the three clades over millions of years of evolution. Also, consistent with previous reports (8), we observed extensive sharing of plasmid replicons between the clades. The plasmids associated with these replicons often carry multiple genes encoding virulence factors or antimicrobial resistance mechanisms, and they readily exchange genetic material between one another (35). The first time detection of *K. variicola* and *K. quasipneumoniae* strains carrying the KPC gene in the United States or *K. variicola* carrying the NDM-1 gene anywhere is particularly concerning for the potential propagation of multidrug resistance and increased virulence capacity among various *Klebsiella* species. Notably, previous studies have suggested that homologous recombination between strains in the different clades does not occur (10). However, strain KPN807, a *K. variicola* isolate, clearly carries a *bla*_OKP_ gene (OKP-B-15) rather than the expected *bla*_LEN_ gene. The chromosomally encoded *bla*_OKP_ gene in strain KPN807 is flanked on both sides by two additional genes with high identity to the homologous region of a *K. quasipneumoniae* reference genome (strain 700603; GenBank accession number CP014696.2). BLAST search of this 7-kb region did not reveal its presence in the genome of any other species or plasmids that could serve as possible third party donors. This 7-kb region of recombination is bordered on one side by the *lacY* and *lacZ* genes, which have been implicated previously in bacterial recombination events (36). Thus, the *K. variicola* isolate KPN807 may have acquired the *bla*_OKP_ gene from *K. quasipneumoniae* by homologous recombination. Similarly, Holt et al. reported one *K. quasipneumoniae* strain that likely underwent a 735-kb homologous recombination with a K. pneumoniae strain (8). Taken together, these data suggest that the possibility of homologous recombination between K. pneumoniae, *K. variicola*, and *K. quasipneumoniae* is underestimated. The extent to which homologous recombination shapes the virulence of these organisms warrants further investigation.

The inability of conventional clinical microbiology laboratory techniques to distinguish *K. variicola* and *K. quasipneumoniae* from K. pneumoniae may contribute to our underestimation of their potential for causing serious human infections (37). Their biochemical phenotypes are closely overlapping. Adonitol fermentation has been suggested to be a useful classifier (K. pneumoniae is reportedly adonitol positive, and *K. quasipneumoniae* and *K*. *variicola* are reportedly adonitol negative), but the phenotype is unstable and not consistent between all strains within the three species (38). Other biochemical tests, such as indole positivity, are similarly flawed (38). The presence of the nitrogen-fixing *nif* operon has been classically associated with agricultural *Klebsiella* isolates; however many of our strains isolated from human infections still carried *nif* genes (8, 39). Misidentification has also propagated into the Klebsiella pneumoniae MLST and other genotyping schema. Many of our *K. variicola* and *K. quasipneumoniae* strains had perfect MLST allele and capsule locus genotype matches within the K. pneumoniae schemes (Table 1). Similarly, K. pneumoniae reference strains Kp5-1 and Kp342 are clearly genomically allied with *K. variicola* strains yet were identified as K. pneumoniae before *K. variicola* was recognized as a distinct species (10). The *K. quasipneumoniae* reference strain 700603 was originally identified as an ATCC-type strain of K. pneumoniae and is still present in the ATCC catalog as a K. pneumoniae subsp. *pneumoniae* (https://www.atcc.org/Products/All/700603.aspx). In addition to type strain misidentification, omission of these pathogens from reference databases may contribute to their misidentification by MALDI-TOF MS methods (15, 24, 40). The 40 strains recovered by our clinical microbiology laboratory, which uses MALDI-TOF MS as the primary identification method for Gram-negative rods, were reported as K. pneumoniae. Although *K. variicola* is included in the current Bruker microorganism reference library (v6.0.0.0), the earlier library versions used when these isolates were recovered in 2011 to 2015 contained at least one *K. variicola* strain misassigned as a K. pneumoniae strain. Consistent with this idea, all *K*. *variicola* strains, except for the outlier KPN1705, were correctly identified when reanalyzed using the current database in 2017. The *Klebsiella* 10982-like strain KPN1705 is still identified as a K. pneumoniae by the current Bruker database (v6.0.0.0), despite its genomic similarity to *K. variicola*. Similarly, *K. quasipneumoniae* is not included in the current Bruker microorganism reference library (v6.0.0.0) or in previous versions. As a direct result, the Bruker MALDI-TOF MS will not identify *K. quasipneumoniae*, and all of these strains are misidentified as K. pneumoniae. As 12 of 95 (12.6%) of our non-ESBL-producing K. pneumoniae strains tested from 2017 were actually *K. quasipneumoniae*, the full extent of *K. quasipneumoniae*-caused human disease may be vastly underestimated. Commercially available molecular methods also misidentify these pathogens as K. pneumoniae or fail to identify them altogether (41, 42). Finally, lab-developed multiplex PCR assays that detect specific core chromosomal beta-lactamases have been cited as potential methods of differentiation (30). Although the *bla*_SHV_, *bla*_OKP_, and *bla*_LEN_ genes are closely associated with the chromosomes of K. pneumoniae, *K. quasipneumoniae*, and *K. variicola*, respectively, our data clearly reveal the presence of *bla*_SHV_ on plasmids in strains belonging to all three clades and one strain that acquired a different chromosomally encoded beta-lactamase gene by homologous recombination.

These data provide new insight into the natural history and pathogenesis of *K. variicola* and *K. quasipneumoniae*, as well as the novel 10982-like *Klebsiella* species. Larger studies using comprehensive population-based strain collections are needed to confirm the extent of potential recombination between these *Klebsiella* and its potential impact on the virulence of these important human pathogens.

## MATERIALS AND METHODS

### Collection of K. pneumoniae strains.

We previously sequenced the genomes of 1,777 ESBL-producing *Klebsiella* strains recovered from patients with infections in our health care system (Houston Methodist Hospital) from 2011 to 2015 (18). To determine the possible presence or absence of *K. variicola* and *K. quasipneumoniae* strains among non-ESBL-producing isolates, we sequenced the genomes of an additional 95 *Klebsiella* strains collected in 2017. All strains were identified as Klebsiella pneumoniae by MALDI-TOF MS. Clinical significance was evaluated by review of the electronic medical record. Strains were cryopreserved at the time of recovery by transferring colonial material to Todd-Hewitt broth containing 20% glycerol and storing at −80°C. This study was approved by the institutional review board at Houston Methodist Hospital and Research Institute (protocol IRB1010-0199).

### Whole-genome sequencing of *Klebsiella.*

To prepare whole-genome sequencing libraries, the cryopreserved stocks were grown on tryptic soy agar containing 5% sheep blood. Genomic DNA was extracted using standard methods (Qiagen, Valencia, CA), and NexteraXT libraries were prepared using the manufacturer’s protocols (Illumina, San Diego, CA) and sequenced on an Illumina MiSeq or NextSeq instrument.

### Bioinformatic analysis of strains.

The single nucleotide polymorphism calling pipeline and additional bioinformatic pipelines were described previously (18). Strains from Holt et al. were downloaded from European Nucleic Acid Archive (accession number ERP000165). BLAST was performed using the NCBI BLAST toolkit as well as CLC Genomics Workbench v.10.1. Visualization of SNP distribution was performed using CLC Genomics Workbench v.10.1. FASTQ files were assembled into contigs using Spades v3.9.0, and contigs were annotated using Prokka v1.12 (43, 44). Gene content analysis was performed using Roary v3.6.1 (45). The 12 strains of ESBL-producing K. pneumoniae included for pangenome comparison are KPN1, KPN2, KPN9, KPN11, KPN12, KPN17, KPN18, KPN133, KPN1998, KPN2000, KPN2108, and KPN2129 (18). Assembly of SNPs into phylogenetic trees was accomplished with the scripts prephix v3.3.0, phrecon v4.6.0, and FastTreeMP v2.1 (46). Prephix and phrecon are available from GitHub (https://github.com/codinghedgehog).

### MALDI-TOF MS identification.

Identification of isolates was performed in the Houston Methodist clinical microbiology laboratory using a Bruker Biotyper MALDI-TOF MS as part of the standard clinical microbiology practice as described previously (19). Briefly, colonial material from agar plates or pelleted cells from liquid blood cultures were transferred to the target plate, dried at room temperature for approximately 2 min, and covered with alpha-cyano-4-hydroxycinnamic acid (HCCA) matrix. Spectra were collected using the default instrument settings and interpreted using the research use only microorganism reference library on the Biotyper (Bruker, Billerica, MA). At the time of isolation in 2011 to 2015, Bruker microorganism reference library versions starting with 4.0.0.0 were used. The strains were reanalyzed in 2017 using version 6.0.0.0, which was installed in January 2017.

### Accession number(s).

The genomes of the strains sequenced for this study have been deposited in the NCBI database under BioProject PRJNA376414 and PRJNA386693.
